# Rituximab-Based Management of Autoimmune Blistering Diseases: A Case Series Highlighting Ocular and Systemic Presentations

**DOI:** 10.7759/cureus.96433

**Published:** 2025-11-09

**Authors:** Sebastián J Vázquez-Folch, Gabriel A Jiménez-Berríos, Victor J Vazquez, Natalio Izquierdo

**Affiliations:** 1 School of Medicine, Universidad Central del Caribe, Bayamón, PRI; 2 Comprehensive Cancer Center, Medical Sciences Campus, University of Puerto Rico, San Juan, PRI; 3 Department of Surgery, School of Medicine, Medical Sciences Campus, University of Puerto Rico, San Juan, PRI

**Keywords:** acantholysis, desmoglein, mucous membrane pemphigoid, pemphigus vulgaris, rituximab

## Abstract

Mucous membrane pemphigoid (MMP), bullous pemphigoid (BP), and pemphigus vulgaris (PV) are rare autoimmune blistering disorders of the skin and mucous membranes that often result in delayed diagnosis and substantial morbidity. MMP is characterized by progressive conjunctival scarring, while BP typically presents with tense cutaneous blisters and PV with intraepidermal acantholysis driven by desmoglein autoantibodies. We present two women, aged 68 and 89 years, with ocular MMP, and one woman with PV, aged 48 years, highlighting the importance of early ophthalmic evaluation and prompt immunosuppressive therapy. Histopathology and immunofluorescence confirmed disease-specific patterns, with MMP demonstrating conjunctival fibrosis and subepithelial blistering, and PV showing vesiculobullous eruptions and antibody-mediated activity. All three patients responded favorably to rituximab, achieving steroid-sparing remission and stable disease control, underscoring its role as a key therapeutic option, particularly in vision-threatening ocular MMP. These findings emphasize the need for timely biopsy, comprehensive antibody testing, and early intervention, while supporting further research to standardize rituximab regimens and optimize long-term outcomes.

## Introduction

Mucous membrane pemphigoid (MMP), bullous pemphigoid (BP), and pemphigus vulgaris (PV) are rare autoimmune blistering disorders of the skin and mucous membranes. These conditions are often underdiagnosed, leading to delayed intervention and significant morbidity [1-3]. MMP, also known as cicatricial pemphigoid, is characterized by progressive scarring, most commonly affecting the conjunctiva, oral cavity, and pharynx [1]. Ocular MMP often presents with chronic conjunctivitis and subepithelial blistering, progressing to conjunctival fibrosis and potential vision loss if untreated [2]. In contrast, PV is an intraepidermal blistering disorder caused by IgG autoantibodies against desmoglein 1 and 3, leading to acantholysis and keratinocyte loss of cohesion [3]. Pemphigoid diseases, including MMP and BP, result from autoantibodies against hemidesmosomal proteins such as BP180 and BP230, producing subepidermal blisters [4,5]. While BP typically presents in elderly patients with pruritic, tense cutaneous blisters and little mucosal involvement, MMP is distinguished by predominant mucosal disease and high risk of ocular complications.

Despite their differences, all three disorders share an autoimmune pathogenesis targeting adhesion proteins essential for epithelial integrity [1,4]. Diagnosis requires integration of clinical features, histopathology, and immunofluorescence testing to detect disease-specific autoantibodies. This case series describes two patients with ocular MMP and one patient with PV, emphasizing the role of ophthalmic evaluation, timely diagnosis, and immunosuppressive therapy (particularly rituximab) in preventing irreversible morbidity.

## Case presentation

We present the clinical characteristics of three non-consecutive patients. They came voluntarily to receive medical care at our private clinic in 2023.

Case 1: Ocular mucous membrane pemphigoid

An 89-year-old woman with a history of right breast invasive ductal carcinoma (estrogen receptor (ER)+, progesterone receptor (PR)+, HER2-) diagnosed in 2011, treated with quadrantectomy, adjuvant, and anastrozole (completed 2018), presented in February 2023 with right ocular redness and mucous membrane changes localized to the inferior fornix. Conjunctival biopsy confirmed MMP.

Her initial treatment regimen was rituximab 375 mg/m² IV weekly × 4 doses alongside IV solumedrol induction (three days). A repeat biopsy in June 2023 showed persistent disease, prompting re-induction with the same regimen, which was completed in August 2023. She subsequently transitioned to rituximab 500 mg IV every six months for maintenance therapy (initiated February 2024, last infusion August 2024), with stable disease.

**Figure 1:**
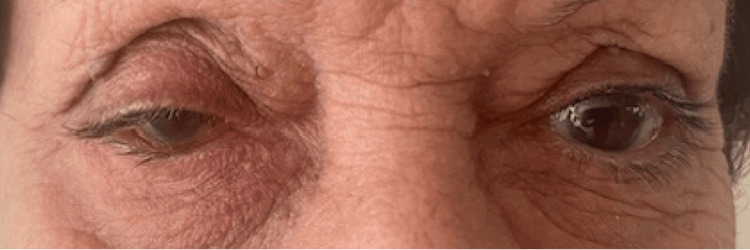
External appearance (Case #1). The patient presented with conjunctival redness, edema, and mild eyelid swelling.

Recent laboratory testing (January 2025) revealed WBC of 8.19 ×10³/µL, RBC of 4.53 ×10⁶/µL, hemoglobin (Hgb) at 13.0 g/dL, hematocrit (Hct) of 41.6%, platelets at 276 ×10³/µL, creatinine at 0.88 mg/dL, and glucose at 98 mg/dL, demonstrating preserved hematologic and renal function. On physical exam (Figure 1), she was in no acute distress. When she came for her appointment, her Eastern Cooperative Oncology Group (ECOG) score was 2 (ambulatory and capable of self-care but unable to carry out work activities). Ophthalmologic exam showed subcorneal blistering of the conjunctiva with redness and drainage in the right eye. No cutaneous lesions, lymphadenopathy, or systemic involvement were noted.

Histopathology of the conjunctival biopsy showed subepithelial blister formation with a smooth epidermal undersurface, pauci-inflammatory lymphocytic infiltrate, and squamous metaplasia (Table 1). The periodic acid-Schiff (PAS) stain was negative for microorganisms or basement membrane thickening. Direct immunofluorescence revealed linear IgG and C3 deposition along the basal epithelial poles in a “capping” pattern, while indirect immunofluorescence detected circulating IgG pemphigoid antibodies, confirming the diagnosis.

**Table 1:** Antibody testing and immunopathologic correlation. IgG: immunoglobulin G; BP: base pair; PV: pemphigus vulgaris; MMP: mucous membrane pemphigoid; OD: right eye; OS: left eye.

Parameter	Patient 1: Ocular mucous membrane pemphigoid (OD)	Patient 2: Cicatricial mucous membrane pemphigoid (OS)	Patient 3: Pemphigus vulgaris
Direct immunofluorescence	Linear IgG + C3 at basal epithelial poles (“capping” pattern)	Linear IgG + C3 at basal epithelial poles (“capping” pattern)	Intercellular IgG + C3 in “fish-net” pattern
Indirect immunofluorescence	Circulating IgG pemphigoid antibodies	Circulating IgG pemphigoid antibodies	Circulating IgG anti-Dsg1 and anti-Dsg3 antibodies
Desmoglein-1	Not tested	<14 (negative)	156 U at diagnosis → normalized (<14) by 2023
Desmoglein-3	Not tested	<9 (negative)	173 U at diagnosis → normalized (<9) by 2023
Other antigen-specific testing (BP180, BP230, laminin 332, type VII collagen, α6β4)	Not performed	Not performed	Not applicable (PV diagnosis)
Correlation with histopathology	Subepithelial blister with lymphocyte-predominant infiltrate; consistent with MMP	Subepithelial blister with lymphocyte-predominant infiltrate; consistent with MMP	Suprabasilar acantholysis; consistent with PV
Clinical relevance	Missing laminin 332 testing: could assess malignancy risk	Missing laminin 332 testing: could assess malignancy risk	Antibody titers paralleled disease activity and treatment response

As of January 2025, she remains on maintenance rituximab with stable ocular disease under close dermatology and ophthalmology follow-up. Given her history of breast carcinoma in remission and advanced age, her management plan emphasizes continued immunosuppressive therapy with rituximab 500 mg IV every six months (next due in February 2025), cautious prednisone 1 mg oral (PO) daily (tapering dose), and ongoing surveillance for breast cancer recurrence.

Case 2: Cicatricial mucous membrane pemphigoid

A 68-year-old female presented in June 2023 with conjunctival hyperemia and inflammation of the left eye. Ophthalmologic evaluation raised concern for cicatricial MMP, and excisional conjunctival biopsy confirmed the diagnosis. Desmoglein 1 and 3 antibody levels were within normal limits, helping to exclude PV.

She received induction therapy with a total of four doses of weekly rituximab 375 mg/m² IV infusions alongside IV solumedrol, completed in October 2023. A trial of dapsone was discontinued due to adverse effects. She subsequently underwent cataract surgery and has since been maintained on rituximab 500 mg IV every six months, with her most recent infusion in October 2024 being well tolerated and associated with clinical improvement. She remains on prednisone 1 mg PO daily (tapering dose) as adjunctive therapy.

Recent labs (November 2024) demonstrated mild anemia (Hgb = 11.7 g/dL, RBC = 3.97 ×10⁶/µL) with otherwise preserved hematologic and metabolic function. An echocardiogram in October 2024 showed an ejection fraction (EF) of 55%. On physical examination, she was in no acute distress and had an ECOG performance status of 1, with left conjunctival scarring and erythema but no systemic lesions or organ involvement.

Histopathology of the conjunctival biopsy revealed a focal subepithelial blister with a smooth epidermal undersurface, pauci-inflammatory lymphocytic infiltrate, and squamous metaplasia (Table 1). Direct immunofluorescence showed linear IgG and C3 deposition along the basal poles of epithelial cells (“capping” pattern), while indirect immunofluorescence detected circulating pemphigoid antibodies, confirming the diagnosis.

As of December 2024, the patient remains clinically stable on rituximab maintenance therapy with ophthalmology follow-up.

Case 3: Pemphigus vulgaris

A 48-year-old female was diagnosed with PV in May 2020 after a biopsy of an upper trunk lesion confirmed the diagnosis. Histopathologic examination of the skin biopsy from the upper trunk demonstrated intraepidermal acantholytic dermatitis with prominent suprabasilar acantholysis, creating a “row of tombstones” appearance in the basal keratinocytes. Direct immunofluorescence revealed intercellular deposition of IgG and C3 throughout the epidermis in a “fish-net” pattern, while indirect immunofluorescence confirmed circulating IgG antibodies against desmoglein-1 and desmoglein-3. Initial serology revealed markedly elevated desmoglein-1 antibodies (156 U) and desmoglein-3 antibodies (173 U), while screening prior to immunosuppressive therapy showed a nonreactive hepatitis profile and negative tuberculin test.

She was treated with rituximab induction therapy of 1000 mg IV every two weeks in two doses in August 2020, followed by re-induction with 500 mg in September 2021, and then maintenance rituximab 500 mg IV six months later. She also received prednisone (20 mg during induction, with tapering thereafter), in combination with diphenhydramine 50 mg/mL as premedication for infusion-related reactions. Over the treatment course, desmoglein antibody titers progressively normalized (Dsg1 < 14 and Dsg3 < 9 by June 2023), correlating with sustained clinical remission.

The patient’s most recent laboratory results in June 2024 demonstrated WBC at 6.4 ×10³/µL, Hgb at 13.6 g/dL, Hct at 45.8%, platelets at 200 ×10³/µL, glucose at 113 mg/dL, lactate dehydrogenase (LDH) at 131 U/L, and erythrocyte sedimentation rate (ESR) at 1 mm/hr, consistent with remission and absence of systemic inflammation. On physical examination in July 2024, she remained off systemic corticosteroids, with no evidence of relapse, and was under dermatology follow-up. She declined influenza and pneumococcal vaccination in July 2024, as well as COVID-19 vaccination. Her social history was notable for being married, living with her spouse and two children, with no history of tobacco, alcohol, or illicit drug use; she had a history of blood transfusion in 2005.

## Discussion

As shown in Table 1, the immunopathologic findings in this series confirmed disease-specific autoantibody profiles consistent with established mechanisms of autoimmune blistering disease. The PV case demonstrated a direct correlation between desmoglein antibody titers and disease activity, a relationship frequently reported in longitudinal PV cohorts where serologic trends parallel clinical remission or relapse [4-7]. In contrast, both ocular MMP cases demonstrated the expected subepithelial blistering and linear IgG/C3 deposition along the epithelial basement membrane, reinforcing that pemphigoid disorders primarily target hemidesmosomal adhesion complexes rather than desmosomal cadherins. The absence of full antigen-specific profiling in these cases differs from current diagnostic recommendations but did not impair diagnostic clarity, as the histopathologic and immunofluorescence patterns remained strongly disease-defining.

Clinical manifestations and disease patterns

The clinical presentations in our MMP patients (conjunctival hyperemia, mucosal changes, and progressive scarring) are consistent with prior reports indicating ocular involvement in up to 70% of MMP cases and its association with irreversible blindness when intervention is delayed [2]. The degree of scarring observed aligns with studies showing that the pace of fibrosis varies based on the timing of diagnosis and initiation of immunosuppression. This supports the widely recognized principle that delayed referral significantly worsens long-term ocular outcomes [8].

In contrast, our PV patient presented with a vesiculobullous eruption of the skin and oral mucosa, which mirrors the typical mucocutaneous distribution seen in over 80% of PV cases [6]. The absence of ocular involvement further aligns with literature noting ocular disease in PV as uncommon (<16%), emphasizing the broader phenotypic divide between PV and the predominantly mucosal scarring phenotype of MMP.

Pathophysiology and histopathology correlation

The histopathologic findings in our cases structurally mirrored their respective disease mechanisms. MMP biopsies demonstrated subepithelial clefting and squamous metaplasia with a lymphocyte-predominant infiltrate, consistent with chronic cicatrizing inflammation described in ocular MMP cohorts. Meanwhile, the PV biopsy displayed intraepidermal acantholysis with suprabasal clefting and the characteristic “fish-net” intercellular IgG pattern, findings that remain pathognomonic for PV. These correlations reinforce the diagnostic importance of pairing histology with direct immunofluorescence to differentiate between superficially similar blistering disorders.

Therapeutic considerations and rituximab’s role

Rituximab has demonstrated superior remission durability in PV compared to conventional immunosuppressants, as confirmed in randomized trials such as PEMPHiX [9]. Our PV patient’s sustained steroid-free remission parallels these findings, supporting rituximab as a first-line agent.

In ocular MMP, rituximab has similarly emerged as an effective therapy, with growing evidence of its ability to halt fibrosis progression and reduce corticosteroid exposure [10]. Both MMP patients in this series responded favorably to rituximab induction and maintenance. While literature describes variable remission durability in MMP compared to PV, our outcomes likely reflect the benefit of relatively early initiation of rituximab prior to advanced conjunctival scarring. Notably, patient 1 had initially been labeled as bullous pemphigoid before biopsy confirmation of ocular MMP, illustrating the well-documented diagnostic overlap within pemphigoid spectrum disorders and the importance of biopsy review in atypical presentations [11].

Importance of early diagnosis

Consistent with prior studies, our outcomes highlight that prompt biopsy and early ophthalmologic involvement are crucial, particularly in ocular MMP, where irreversible fibrosis may occur before symptoms are recognized [2,8]. The favorable disease control observed here supports the growing consensus that early biologic therapy can alter disease trajectory.

Limitations and future directions

This series is limited by the absence of extended antigen-specific antibody testing (e.g., BP180, BP230, laminin 332, and type VII collagen). Laminin 332 reactivity, in particular, carries prognostic implications regarding malignancy risk and would have been clinically relevant in our patient with prior breast cancer. Additionally, the small sample size and variable follow-up durations limit generalizability. These limitations reflect ongoing challenges across the field, as multicenter longitudinal data remain limited. Future work should prioritize standardized rituximab protocols, integration of comprehensive antigen testing, and prospective monitoring to better define remission durability and optimize treatment algorithms.

## Conclusions

Autoimmune blistering diseases, such as MMP and PV, carry substantial morbidity, particularly when ocular involvement threatens irreversible vision loss. This case series illustrates how early biopsy, immunopathologic confirmation, and timely initiation of rituximab can alter the disease course, leading to steroid-sparing remission. Multidisciplinary collaboration between ophthalmology, dermatology, and other specialists remains essential for optimal care. Further research is needed to standardize rituximab regimens and refine long-term management strategies in autoimmune blistering diseases.
